# Impact of chemical snail control on intermediate host snail populations for urogenital schistosomiasis elimination in Pemba, Tanzania: findings of a 3-year intervention study

**DOI:** 10.1186/s13071-024-06565-2

**Published:** 2024-11-26

**Authors:** Lydia Trippler, Said Mohammed Ali, Msanif Othman Masoud, Zahor Hamad Mohammed, Amour Khamis Amour, Khamis Rashid Suleiman, Shaali Makame Ame, Fatma Kabole, Jan Hattendorf, Stefanie Knopp

**Affiliations:** 1 Swiss Tropical and Public Health Institute, Allschwil, Switzerland; 2 University of Basel, Basel, Switzerland; 3 Public Health Laboratory-Ivo de Carneri, Chake-Chake, Pemba United Republic of Tanzania; 4 Department of Preventive Services and Health Education, Zanzibar Ministry of Health, Chake-Chake, Pemba United Republic of Tanzania; 5 Neglected Diseases Program, Zanzibar Ministry of Health, Chake-Chake, Pemba United Republic of Tanzania; 6 Neglected Diseases Program, Zanzibar Ministry of Health, Zanzibar Town, Unguja United Republic of Tanzania

**Keywords:** *Bulinus*, Control, Elimination, Molluscicide, Niclosamide, *Schistosoma*, Snail, Tanzania

## Abstract

**Background:**

The World Health Organization (WHO) has set the goal of eliminating schistosomiasis as a public health problem globally by 2030 and to interrupt transmission in selected areas. Chemical snail control is one important measure to reduce transmission and achieve local elimination. We aimed to assess the impact of several rounds of chemical snail control on the presence and number of the *Schistosoma haematobium* intermediate snail host (*Bulinus* spp.) in water bodies (WBs) on Pemba Island, Tanzania, a setting targeted for elimination of urogenital schistosomiasis.

**Methods:**

During the three annual intervention periods of the SchistoBreak study implemented in the north of Pemba from 2020 to 2024, malacological surveys were conducted up to four times per period in WBs of hotspot implementation units (IUs). Present freshwater snail species, vegetation, and WB characteristics were recorded. If *Bulinus* were found, the snails were inspected for *Schistosoma* infection and snail control with niclosamide was conducted.

**Results:**

Across the three intervention periods, a total of 112 WBs were identified in 8 hotspots IUs. The spatial distribution of WBs with *Bulinus* per IU was heterogeneous, ranging from 0.0% (0/15) of WBs infested in one IU in 2022 to 80.0% (8/10) of WBs infested in one IU in 2021. *Bulinus* presence was significantly associated with lower pH values in WBs (odds ratio: 0.2, 95% confidence interval 0.1–0.4). A total of 0.2% (6/2360) of collected *Bulinus* were shedding *Schistosoma* cercariae. Following snail control, the number of *Bulinus* decreased or remained absent in 56.7% (38/67) of visits at WBs when compared with the previous visit in 2021, 54.9% (28/51) in 2022, and 33.3% (32/96) in 2023. In a total of 43.1% (22/55) of initially infested WBs, no *Bulinus* were found in the survey round conducted a few weeks after the first application of niclosamide. However, 25.4% (14/55) of WBs showed a pattern of recurring *Bulinus* presence.

**Conclusions:**

The distribution of WBs containing *Bulinus* was very heterogeneous. The percentage of *Bulinus* with patent *Schistosoma* infection in our study area was extremely low. Repeated niclosamide application reduced the number of *Bulinus* in WBs, but snails often recurred after one or multiple treatments. While chemical mollusciciding can reduce snail numbers, to fully break the *S. haematobium* transmission cycle, timely diagnosis and treatment of infected humans, access to clean water, sanitation, and health communication remain of prime importance.

*Trial registration*: ISRCTN, ISRCTN91431493. Registered 11 February. 2020, https://www.isrctn.com/ISRCTN91431493

**Graphical Abstract:**

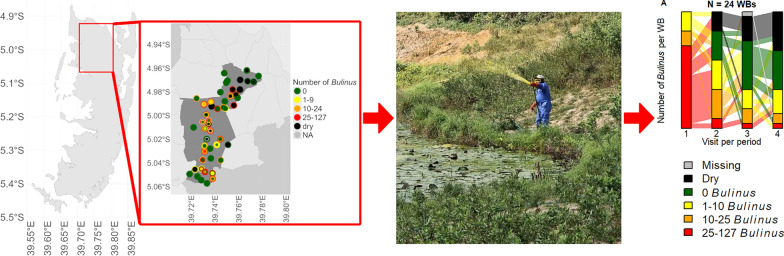

**Supplementary Information:**

The online version contains supplementary material available at 10.1186/s13071-024-06565-2.

## Background

Schistosomiasis is a neglected tropical disease that is prevalent in 78 countries worldwide, with a particularly high prevalence in sub-Saharan African countries [1]. The infective agent is a parasitic blood fluke of the genus *Schistosoma* spp., which is transmitted in a human–snail–human life cycle [2]. The intermediate hosts of the African *Schistosoma* species are freshwater snails of different parasite-specific genera, which thrive in natural freshwater bodies, have a reproduction optimum at 25 °C, and can survive dry periods by estivating around the margins of water bodies [3, 4].

The World Health Organization (WHO) has set the goal of eliminating schistosomiasis as a public health problem globally and to interrupt *Schistosoma* transmission in selected areas by 2030 [1, 2]. To achieve this goal, the WHO recommends the implementation of interventions that address the human–snail–human life cycle at different stages simultaneously [5]. These interventions encompass a number of different strategies, including mass drug administration (MDA) with praziquantel of populations at risk of *Schistosoma* infection, behavior change communication measures, improvements in access to water, sanitation, and hygiene, and chemical snail control with the molluscicide niclosamide [5, 6].

Chemical snail control is recommended as a measure to reduce the intermediate host snail populations [6]. Since niclosamide is not only harmful to snails but may also cause damage to the environment and harms other invertebrates, amphibians, and fish, it should only be applied focally or in specific seasons [6]. Its application is particularly recommended for persistent hotspots of *Schistosoma* transmission, where several rounds of MDA have not resulted in a significant decrease of infection prevalence [5, 6]. Moreover, in combination with other interventions, snail control is expected to accelerate schistosomiasis elimination [5].

Schistosomiasis research, including many malacological and parasitological studies, has a century-long history on the Zanzibar Islands of the United Republic of Tanzania [7]. Studies showed that the only autochthonously transmitted *Schistosoma* species in Zanzibar infecting humans is *S. haematobium* and that the main, and likely only, freshwater snail species responsible for its transmission is *Bulinus globosus* [8–10]. Equally long is the history of interventions to mitigate urogenital schistosomiasis on the islands, including different approaches for snail control [11]. In 2010, the overall *S. haematobium* prevalence on the islands was < 10%, and efforts to eliminate schistosomiasis as a public health problem and to interrupt transmission started with the Zanzibar Elimination of Schistosomiasis Transmission (ZEST) project in 2012 [12]. For the first time in Zanzibar, in randomized areas, large-scale snail control with niclosamide was conducted in addition to biannual MDA, but no added impact was identified owing to very low numbers of infected individuals at the end of the trial in 2017 [12, 13]. In the subsequent SchistoBreak study (2020–2024), snail control was applied in remaining hotspot areas in the north of Pemba Island, together with regular MDA and behavior change communication measures to advance elimination [14].

The research presented here covers results from malacological surveys and mollusciciding interventions conducted in the hotspot areas of the SchistoBreak study. Our aim was to assess the impact of several rounds of chemical snail control on the presence and number of *Bulinus* in targeted water bodies.

## Methods

### Study setting

The SchistoBreak study was conducted in the north of Pemba, one of the two main islands of the Zanzibar Archipelago. The island is located around 30 km off the Tanzanian mainland and is divided into four districts, which are subdivided into 129 small administrative areas, called shehias [15]. The SchistoBreak study was implemented in the districts Micheweni and Wete in 20 shehias, referred to hereinafter as implementation units (IUs) [14]. Implementation units with *S. haematobium* prevalence ≥ 3.0% in an annual school-based parasitological survey or ≥ 2.0% in an annual household-based parasitological survey were considered to be hotspot areas [14, 16]. In these hotspot areas, a combined intervention package consisting of annual school-based and community-based MDA, behavior change communication measures, and snail control were implemented to accelerate urogenital schistosomiasis elimination [14].

### Malacological surveys and snail control

The locations of freshwater bodies in the SchistoBreak study area were mainly identified through an initial survey of IU characteristics, conducted at the onset of the study in 2020 [16]. Additional water bodies and their locations were pointed out by children who were part of surveillance-response interventions in low-prevalence IUs [17]. To decide whether a waterbody in a hotspot IU should be treated with niclosamide, malacological surveys were conducted to investigate whether *Bulinus* were present [14]. To conduct the malacological surveys, two experienced field workers entered the water with protective gear and searched the shoreline for freshwater snails in a 20-m distance for 10 min. The snails were identified morphologically at genus level and the numbers were recorded with a predesigned questionnaire, using the software Open Data Kit (ODK, www.opendatakit.org), installed on Samsung Galaxy Tab A tablets. All collected snails were placed back into the water, with the exception of *Bulinus*, which were transported in plastic containers containing water from the water body to the Public Health Laboratory–Ivo de Carneri (PHL-IdC) in Chake Chake. Here, the snails were kept overnight at room temperature and examined for cercariae shedding the following day. For this purpose, each snail was placed in a single well of a multiple-well plate and covered with drinking water. The plate was placed in direct sunlight as shedding is stimulated by light [18]. After 30 min, each well was inspected for *Schistosoma* cercariae using a dissection microscope. Subsequently, the snails were placed back in the sunlight and wells were inspected again for cercariae after 15 min.

In addition, at the waterbodies, the type of water body (stream/river, pond/lake), temperature, pH value, and conductivity of the water were measured using a water meter (HI98129 Kombi-Tester; Hanna Instruments Deutschland GmbH, Vöhringen, Germany), and information about size, depth, sediment, steepness of shorelines, rice cultivation, vegetation growth, and whether the water body was permanent was recorded in ODK.

Snail control using the molluscicide niclosamide (WP83,1; Bayer AG Crop Science Division, Monheim, Germany) was conducted at human water contact sites at all freshwater bodies in a hotspot IU where *Bulinus* were found, in the current malacological survey, and subsequently at any previous visit during the SchistoBreak study, and additionally, if data from the ZEST study had indicated an infestation with *Bulinus* in the past. The nature and size of the freshwater body dictated the machine with which niclosamide was applied. At small water bodies, plastic backpack sprayers (Farmate, Taizhou Sunny Agricultural Machinery Co., LTd, Taizhou, China) were used; at larger water bodies, a gasoline-powered sprayer (Zhejiang O O Power Machinery Co., Ltd, Zhejiang, China) was applied. The initial concentration of niclosamide in water was 8–10 g of wettable powder dissolved in 1 L of water taken from the water body. One backpack sprayer can hold an initial volume of 10 L of water, in which 80 g of niclosamide was dissolved. The barrel attached to the gasoline-powered sprayer can hold an initial volume of 100 L of water, in which 1000 g of niclosamide was dissolved. The amount of niclosamide and the machines used for each application were recorded in ODK, as well as the area in square meters that was sprayed.

Every water body in a hotspot IU was visited, surveyed, and potentially sprayed with niclosamide up to four times per intervention period of the SchistoBreak study, which covered the months of May–November in each of 2021, 2022, and 2023.

### Data management and statistical analyses

The data collected in ODK were sent to a secured specific ODK server at the Swiss Tropical and Public Health Institute in Allschwil, Switzerland. Subsequently, the data were cleaned with STATA/IC 16.1 (StataCorp LLC, College Station, TX, USA) and analyzed using R version 4.3.2 (www.rproject.org).

To assess the association between environmental factors or the presence of other snail genera with the presence of *Bulinus*, univariable and multivariable logistic regressions were conducted and odds ratios (ORs) with 95% confidence intervals (CIs) were determined. All naïve water bodies in 2021–2023, where snail control had not been previously conducted during the SchistoBreak study, were included in the models. Stepwise selection was used to develop the model of the multivariable regression. Variables with a generalized variance inflation factor > 10 were excluded from the multivariable regression owing to multicollinearity.

The geolocation of water bodies where malacological surveys were conducted was mapped. The number of *Bulinus* found during each visit was stratified into the following categories: zero, 1–9, 10–24, and 25–127. Furthermore, it was indicated whether water bodies were dry when visited. Water bodies overlapping on the map were jittered manually.

To assess the overall change in *Bulinus* numbers per intervention period, the number of *Bulinus* per water body, visit, and intervention period, respectively, was determined for all water bodies where niclosamide was applied at every visit during the intervention period. Hence, the analysis followed a longitudinal design, examining the same water bodies during each intervention period. Water bodies that were already subjected to snail control in intervention period 1 were excluded from the analysis in intervention period 2. Moreover, it was assessed after how many rounds of niclosamide application zero *Bulinus* were found, and whether *Bulinus* reoccurred, i.e., when a water body showed a positive–negative–positive pattern on visit 1–3 or in visit 2–4 despite repeated snail control.

To show the change of *Bulinus* numbers from one visit to the next visit at the same water body after the application of niclosamide, scatterplots were created for all water bodies, where snail control was conducted after the malacological survey of the previous visit and that were not dry on any of the compared visits. To visualize the change, a diagonal with a slope of 1 was inserted. Here, the analysis did not follow a longitudinal approach but water bodies were analyzed from one visit to the next visit. Water bodies that were already subjected to snail control in intervention period 1 were excluded from intervention period 2. Success was defined as either a reduction in snail numbers from one visit to the next or as maintaining zero snails.

## Results

### Water bodies with freshwater snails per year

In the SchistoBreak study, five IUs were considered hotspot IUs in 2021, four in 2022, and three in 2023 (Table 1). In total, there were eight different hotspot IUs identified across the project time. In the three intervention periods, 50 water bodies were visited up to four times in hotspot IUs in 2021, 59 in 2022 and 57 in 2023. In total, 112 different water bodies were visited up to four times across the three periods. Among the 112 water bodies, 97 (86.6%) were streams, 7 (6.3%) were ponds, and 2 (1.8%) were of other types.

**Table 1:** Water bodies with freshwater snails

Year	IU	Visit	WBs visited	Stream	Pond	Other	Dry	WBs with *Bulinus*	*Bulinus* subjected to shedding	*Bulinus* shedding cercariae	WBs with *Cleopatra*	WBs with *Lanistes*	WBs with *Lymnaea*	WBs with *Pila*	WBs with *Thiara*	WBs sprayed	Niclosamide used (g), median (min–max)
2021	5	1	48	43	0	2	3	26	1044	2	0	28	5	0	5	28	200 (80–3000)
		2	50	38	1	1	10	16	205	1	0	20	2	0	2	25	160 (80–2820)
		3	48	36	0	1	11	10	125	0	0	12	3	0	1	23	240 (80–3000)
		4	50	33	0	1	16	8	72	1	0	9	0	0	2	22	160 (80–1500)
2022	4	1	58	48	6	0	4	15	253	0	0	37	0	0	0	19	320 (80–2500)
		2	58	46	4	0	8	12	122	0	0	24	0	0	0	20	400 (80–1500)
		3	59	41	2	0	16	10	80	0	0	30	2	0	0	21	240 (80–1160)
		4	59	21	0	0	38	6	109	0	0	9	1	0	0	13	320 (80–1320)
2023	3	1	57	53	4	0	0	18	274	0	0	33	0	0	0	33	160 (80–1500)
		2	53	48	4	0	1	10	166	0	0	32	1	0	0	33	160 (80–1500)
		3	56	47	3	0	6	17	180	2	0	32	4	0	0	32	160 (160–2000)
		4	56	52	4	0	0	13	0	0	0	30	1	0	0	35	240 (80–1000)

Number of water bodies (WBs) with freshwater snails in hotspot implementation units (IUs) of the SchistoBreak study area in the north of Pemba, Tanzania, in 2021, 2022 and 2023

In the study area, across the three intervention periods, water bodies were found to be infested with freshwater snails of the genera *Bulinus*, *Lanistes*, and *Lymnaea*. The genus *Thiara* was only found in 2021. No water body was found to be infested with *Cleopatra* or *Pila*. Prior to the implementation of snail control, during the initial visit of each intervention period, 54.2% (26/48) of the water bodies in 2021 were found to be infested with *Bulinus*, 25.9% (15/58) in 2022, and 31.6% (18/57) in 2023. Across all intervention periods, a total of 2360 *Bulinus* were collected at water bodies and subjected to examination for the presence of cercariae. Only 0.2% (6/2360) of the *Bulinus* were shedding cercariae.

### Environmental factors associated with *Bulinus* presence in untreated water bodies

**Fig. 1:**
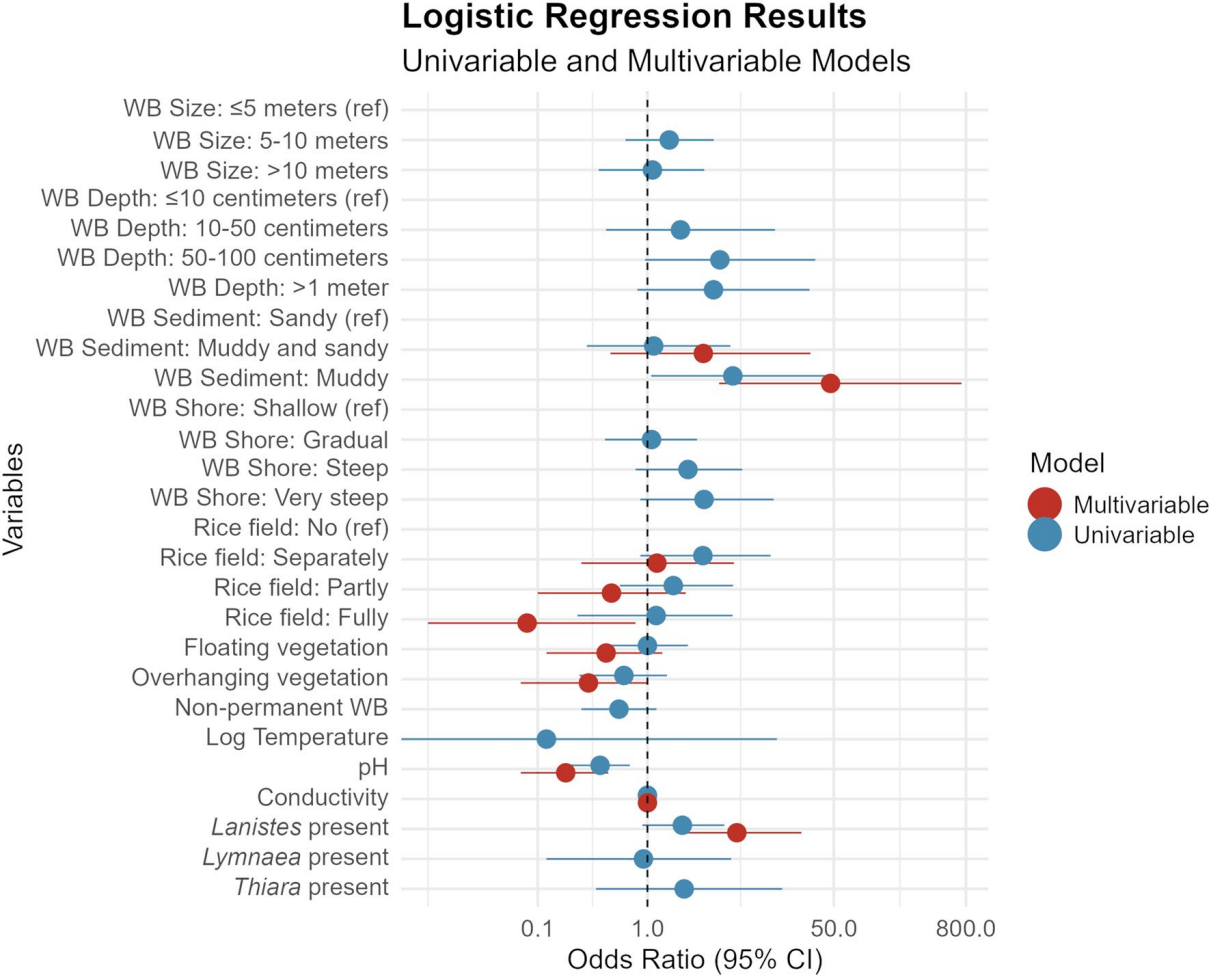
Environmental predictor variables for *Bulinus* presence. Environmental predictor variables for *Bulinus* presence at water bodies (WBs) that were not (yet) sprayed with niclosamide in the north of Pemba, Tanzania

At the initial visit of water bodies, before any of them had been treated with niclosamide, the odds of finding *Bulinus* were significantly higher in water bodies with muddy sediment (multivariable OR: 46.6, 95% CI 4.5–729.5, *P* < 0.01) than in water bodies with sandy sediment only (Fig. 1; Supplementary file 1). Furthermore, the odds of finding *Bulinus* were lower in water bodies that were a rice field (multivariable OR: 0.08, 95% CI 0.01–0.8) compared with water bodies where no irrigation farming was conducted. *Bulinus* presence was significantly associated with lower pH values in both the univariable and the multivariable model (multivariable OR: 0.2, 95% CI 0.07–0.4, *P* < 0.001). In the multivariable model, the presence of *Bulinus* was significantly associated with the presence of snails of the genus *Lanistes* (multivariable OR: 6.5, 95% CI 2.0–25.4, *P* < 0.01).

### Water bodies with *Bulinus* per implementation unit and year

**Fig. 2:**
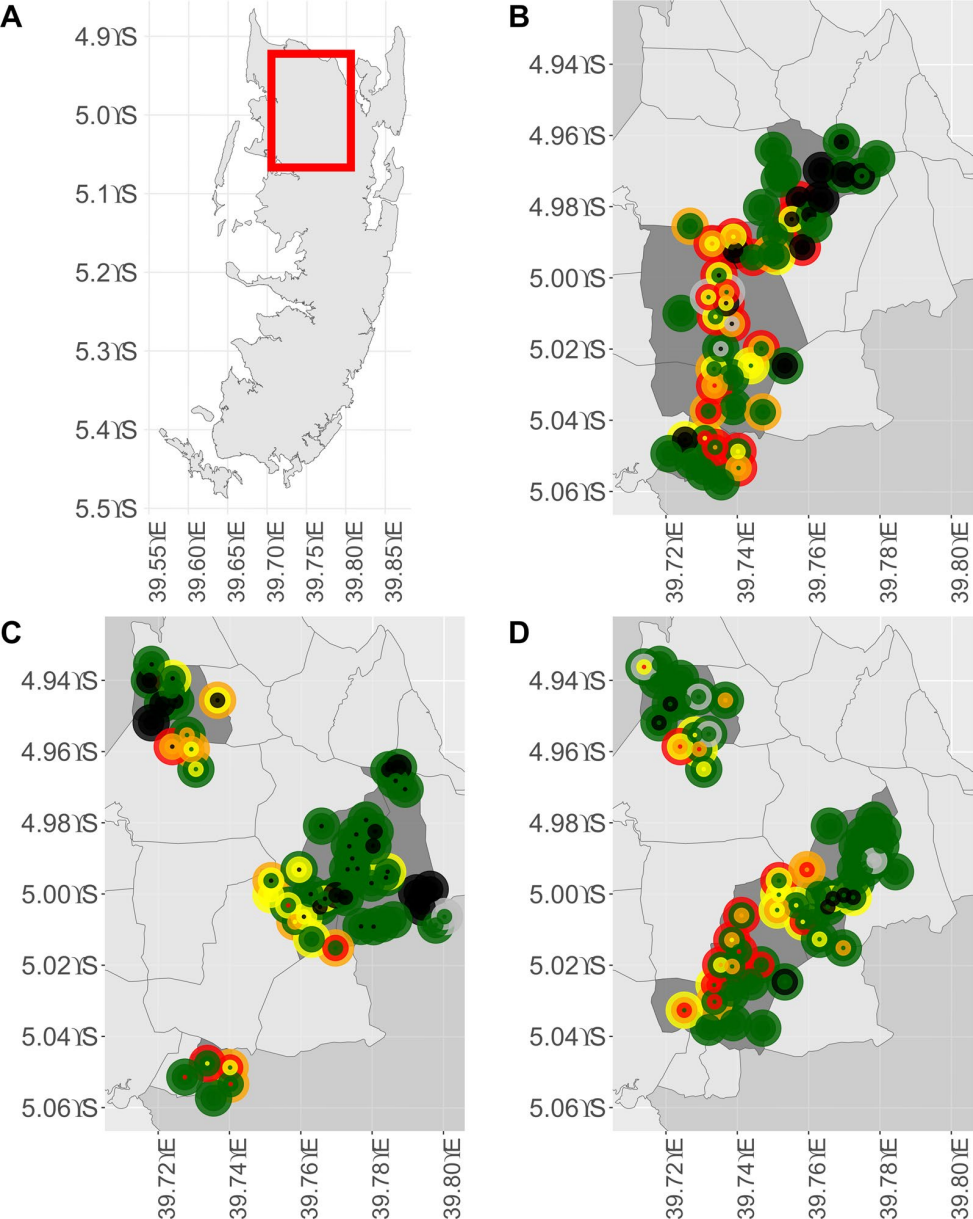
Water bodies with *Bulinus* in the study area. Schistosomiasis hotspot areas in the north of Pemba (**A**) and the presence and number of *Bulinus* in water bodies in 2021 (**B**), 2022 (**C**), and 2023 (**D**). Visits 1–4 from outside to inside circle. To optimize visualization, the locations of overlapping water bodies have been manually jittered. Number of *Bulinus*: green: 0; yellow: 1–9; orange: 10–24; red: 25–127; black: dry; grey: not assessed

In 2021, at the initial visit, the proportion of water bodies with *Bulinus* per IU ranged from 29.4% (5/17) in one IU to 80.0% (8/10) in another IU (Fig. 2B). A total of 50 water bodies were visited up to four times throughout the intervention period, and 56.0% (28/50) of these were found to be infested with *Bulinus* at least once during the visits.

In 2022, the proportion of water bodies with *Bulinus* per IU ranged from 0.0% (0/15) to 66.7% (4/6) at the initial visit Fig. 2C. During the intervention period, a total of 59 water bodies were visited up to four times, and in 35.6% (21/59), *Bulinus* were found on at least one visit Fig. 2D.

In 2023, during the first visit, the proportion of water bodies with *Bulinus* per IU ranged from 18.8% (3/16) to 56.2% (9/16). A total of 57 water bodies were visited up to four times throughout the intervention period, and 43.9% (25/57) harbored *Bulinus* on at least one visit (Fig. 2D). 

Throughout all three intervention periods, 48.2% (54/112) of the water bodies were dry during at least one visit and hence temporary, while 51.8% (58/112) were never dry and hence permanent. Overall, in 33.3% (18/54) of the temporary water bodies, *Bulinus* were found at least once. In 22.2% (12/54) of the temporary water bodies, ten or more *Bulinus* were found at least once. Conversely, in 60.3% (35/58) of the permanent water bodies, *Bulinus* were found at least once, and in 46.6% (27/58) ten or more *Bulinus* were found at least once.

### Change in number of *Bulinus* after mollusciciding, per intervention period

**Fig. 3:**
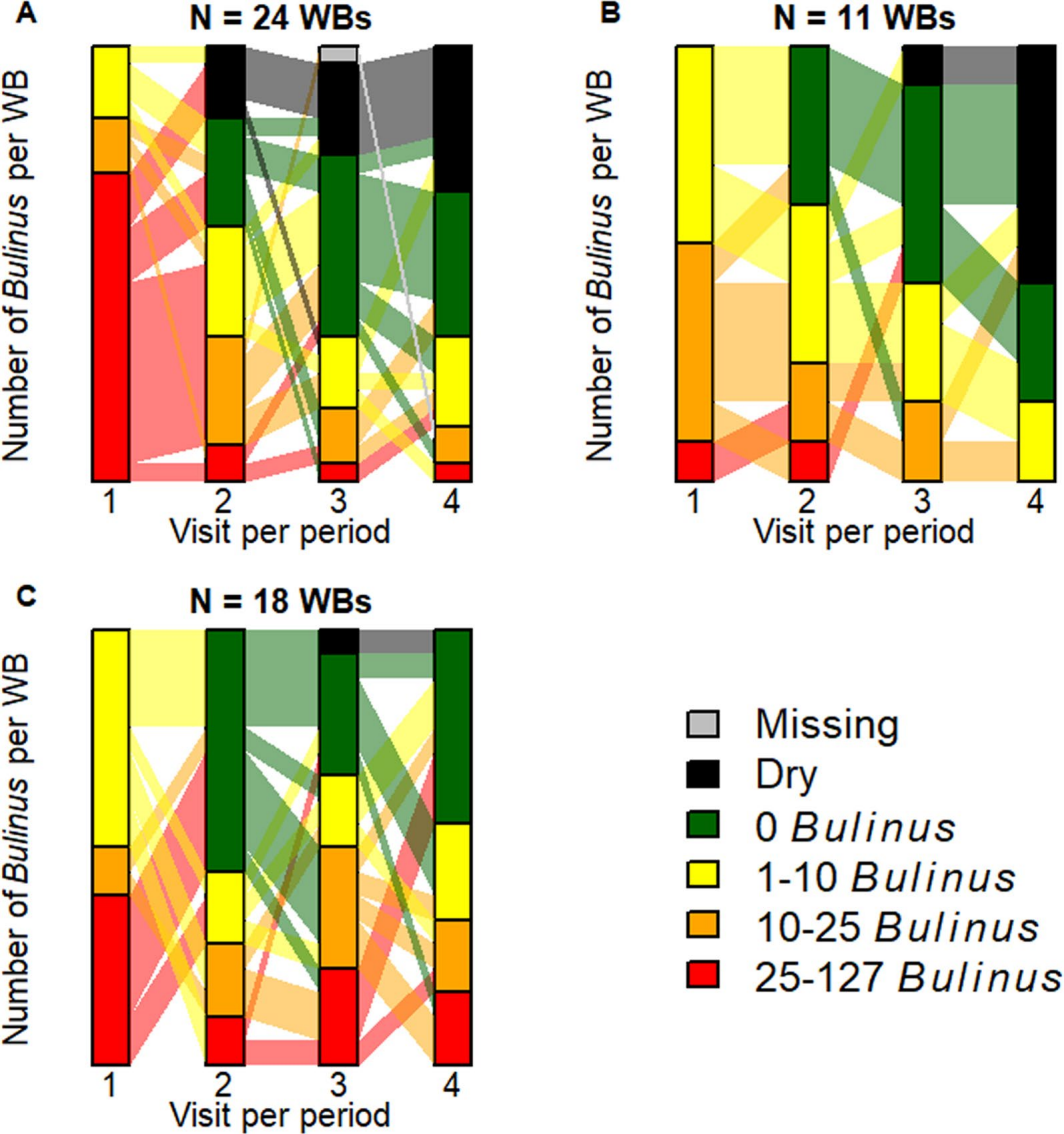
Number of *Bulinus* after snail control. Number of *Bulinus* in water bodies (WBs) that contained *Bulinus* at the initial visit and received snail control at least once during the intervention periods in 2021 (**A**), 2022 (**B**), or 2023 (**C**)

The following paragraphs describe the change in the total number of *Bulinus* and different categories of *Bulinus* numbers, respectively, in water bodies that were infested at the initial visit of the intervention period and hence treated with niclosamide at the initial and all subsequent visits of the respective intervention period unless a water body was dry (Fig. 3 A-C).

#### Intervention period 1 (2021)

During the first visit in the first intervention period, a total of 1006 *Bulinus* were found in 26 water bodies, which were subsequently treated with niclosamide. Across the intervention period, 49,380 g of niclosamide was used to spray 16,875 m of shoreline. By the last visit, the number of *Bulinus* was reduced by 92.8% to 72 *Bulinus* found in 16 water bodies. A total of 29.2% (7/24) of the water bodies exhibited a consistent decline in the number of *Bulinus* from higher to lower categories or remained at zero from one visit to the next (Fig. 3A). Another 70.8% (17/24) of the water bodies showed oscillating patterns between the categories, and no water body exhibited a consistent increase in the categories. Finally, in 20.8% (5/24) of the water bodies, *Bulinus* reoccurred in visit 3 or 4, while no *Bulinus* had been found during visit 2 or 3, respectively.

In the same 24 water bodies, 296 snails of the genus *Lanistes* were found during the first visit. By the last visit, the number of *Lanistes* was reduced by 87.8% to 36 *Lanistes* found in 16 water bodies.

#### Intervention period 2 (2022)

During the first visit in the second intervention period, a total of 133 *Bulinus* were found in 11 water bodies that were not dry and had not already been visited in the first intervention period. The 11 water bodies were subsequently subjected to snail control. Across the intervention period, 39,860 g of niclosamide was used to spray 10,092 m of shoreline. By the last visit, the number of *Bulinus* had decreased by 95.5% to a total of six *Bulinus* in five water bodies. A total of 27.3% (3/11) of the water bodies exhibited a consistent decline in the number of *Bulinus* from higher to lower categories or remained at zero from one visit to the next (Fig. 3B). Another 72.7% (8/11) of the water bodies showed oscillating patterns within the categories. No water body exhibited a consistent increase in the categories. In a total of 9.1% (1/11) of the water bodies, *Bulinus* reoccurred in visit 3 or 4, while no *Bulinus* had been found during visit 2 or 3, respectively.

In the same 11 water bodies, 108 snails of the genus *Lanistes* were found during the first visit. By the last visit, the number of snails was reduced by 77.8% to 24 *Lanistes* found in 5 water bodies.

#### Intervention period 3 (2023)

During the first visit in the last intervention period, a total of 274 *Bulinus* were found in 18 water bodies, which were subsequently sprayed with niclosamide. Across the intervention period, 50,840 g of niclosamide was used to spray 17,639 m of shoreline. By the last visit, the number of *Bulinus* had decreased by 36.1% to a total of 175 *Bulinus* in these water bodies. A total of 66.7% (12/18) of the water bodies exhibited a consistent decline in the number of *Bulinus* from higher to lower categories or remained at zero from one visit to the next (Fig. 3C). Another 33.3% (6/18) of the water bodies showed oscillating patterns within the categories, and no water body exhibited a consistent increase in the categories. In a total of 38.9% (7/18) of the water bodies, *Bulinus* reoccurred in visit 3 or 4 after zero *Bulinus* had been found during visit 2 or 3, respectively.

In the same 18 water bodies, 199 *Lanistes* were found during the first visit. By the last visit, the number of *Lanistes* was reduced by 68.8% to 62 snails.

### Change in number of *Bulinus* after mollusciciding, per visit and intervention period

**Fig. 4:**
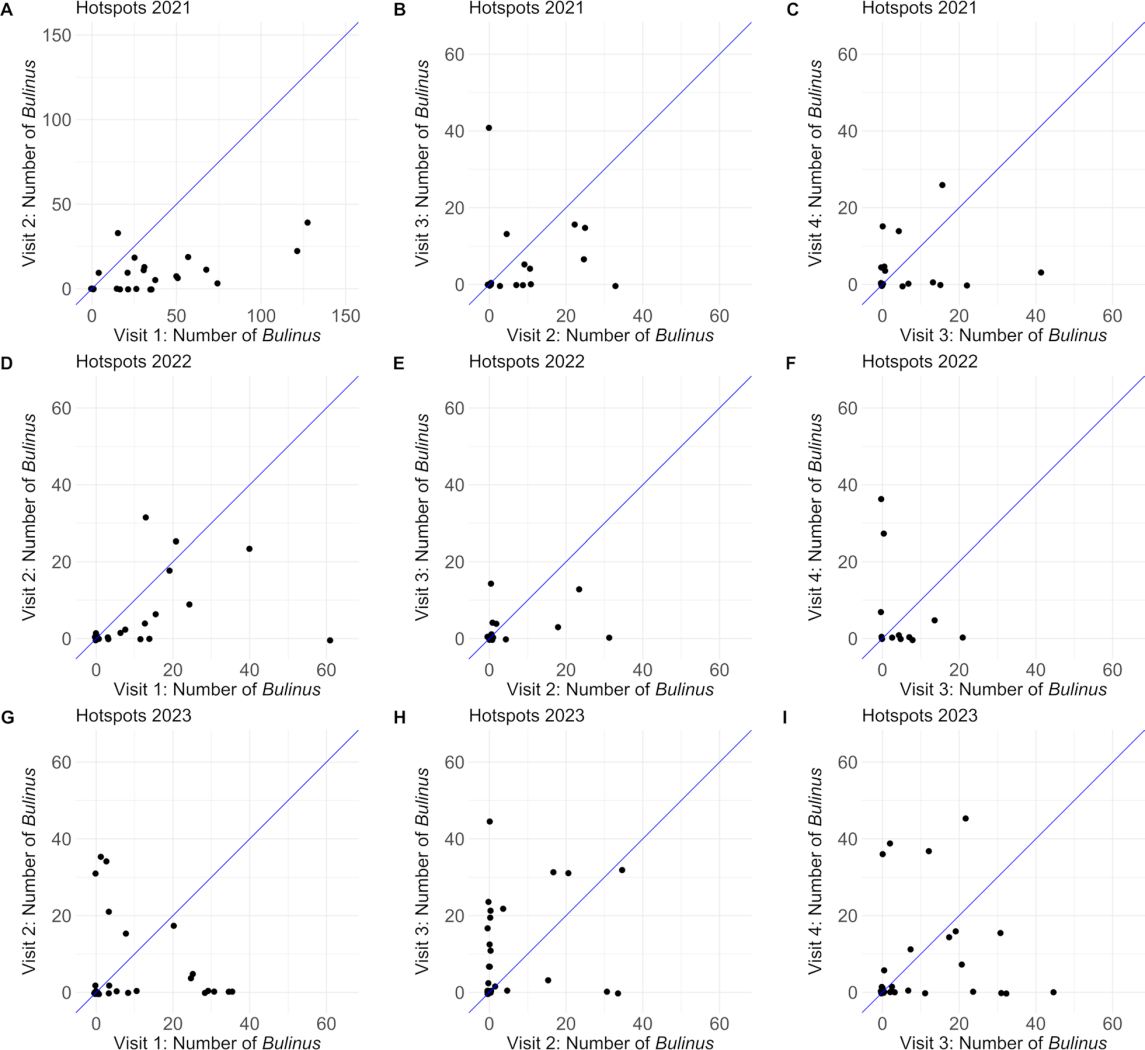
Number of *Bulinus* in water bodies before and after mollusciciding. Number of *Bulinus* in water bodies in hotspot areas per visit (1–4) and per intervention period (2021–2023). Between the compared visits, niclosamide was applied to the water bodies. To optimize visualization, the axes of the first figure (**A**) range from 0 to 150 while the axes of the other figures (**B**–**I**) range from 0 to 60. Jittering was used to allow all points to be shown

The following paragraphs describe the change in the number of *Bulinus* in infested water bodies per visit after niclosamide was applied in the previous visit (Fig. 4A–I).

#### Intervention period 1 (2021)

In 2021, a total of 24 water bodies were treated with niclosamide at visit 1. Comparing the number of *Bulinus* found at visit 1 and visit 2, in 83.3% (20/24) of the water bodies, the number of *Bulinus* decreased or remained zero. From visit 2 to visit 3, *Bulinus* numbers were reduced or remained zero in 54.5% (12/22) of previously treated water bodies. From visit 3 to 4, *Bulinus* numbers had decreased or remained zero in 28.6% (6/21) of previously treated water bodies. Overall, in the intervention period of 2021, the number of *Bulinus* decreased or remained zero in 56.7% (38/67) of the comparisons of *Bulinus* numbers from current to previous visit.

#### Intervention period 2 (2022)

In 2022, a total of 19 water bodies were treated with niclosamide at the first visit. From the first to the second visit, in 68.4% (13/19) of the water bodies, the number of *Bulinus* decreased or remained zero. From visit 2 to visit 3 and from visit 3 to 4, *Bulinus* numbers in 42.1% (8/19) and 53.8% (7/13) of the water bodies decreased or remained zero, respectively. Overall, in the intervention period of 2022, the number of *Bulinus* decreased or remained zero in 54.9% (28/51) of the comparisons of *Bulinus* numbers from the current to the previous visit.

#### Intervention period 3 (2023)

In 2023, a total of 42.4% of the water bodies were treated with niclosamide at the first visit. The comparison of the first with the second visit showed that, in 42.4% (14/33) of the water bodies, the number of *Bulinus* was reduced or remained zero. From visit 2 to visit 3, *Bulinus* numbers decreased or remained zero in 16.1% (5/31) of previously treated water bodies. From visit 3 to 4, *Bulinus* numbers decreased or remained zero in 40.6% (13/32) of previously treated water bodies. Overall, in the intervention period of 2023, the number of *Bulinus* decreased in 33.3% (32/96) of the comparisons of *Bulinus* numbers from the current to the previous visit.

### Water bodies with a reduction of *Bulinus* to zero across visits and intervention periods

The following paragraph describes the change of the number of water bodies infested with *Bulinus* from the first to the fourth visit, when water bodies were treated with niclosamide on all visits.

Throughout all three intervention periods, there were 55 water bodies that contained *Bulinus* at the first visit and where snail control was subsequently conducted on all following visits (Table 2). Among them, 51 were not dry during the second visit, and 43.1% (22/51) of these water bodies had zero *Bulinus* at this second visit. At the third visit, 45.7% (21/46) of the water bodies that were not dry had zero *Bulinus*. At the fourth visit, 47.4% (18/38) of the water bodies that were not dry had zero *Bulinus*. A total of 10.9% (6/55) of the water bodies never reached zero *Bulinus*, despite the consecutive application of niclosamide.

**Table 2:** Water bodies with a reduction of *Bulinus* to zero

Year	Visit 1: WBs with *Bulinus*	Visit 2: WBs with no *Bulinus*	Visit 3: WBs with no *Bulinus*	Visit 4: WBs with no *Bulinus*	Never zero *Bulinus*
2021	26	36.4% (8/22)	57.9% (11/19)	50.0% (8/16)	11.5% (3/26)
2022	11^a^	36.4% (4/11)	50.0% (5/10)	60.0% (3/5)	9.1% (1/11)
2023	18	55.6% (10/18)	29.4% (5/17)	41.2% (7/17)	11.1% (2/18)
Total	55	43.1% (22/51)	45.7% (21/46)	47.4% (18/38)	10.9% (6/55)

Number of water bodies (WBs) with *Bulinus* at first visit and water bodies without *Bulinus* after snail control at subsequent visits

^a^Difference to map where *N* = 13: the map includes all water bodies surveyed. Two among the 13 water bodies had been treated with niclosamide in 2021 already and were hence excluded from the table in 2022

## Discussion

The elimination of schistosomiasis as a public health problem globally and the interruption of *Schistosoma* transmission in selected areas are goals defined by the WHO for 2030 [1, 2]. Chemical snail control is recommended as a measure to diminish the intermediate host snail populations and, in combination with other interventions, is expected to reduce transmission significantly and thus to hasten elimination [5, 6]. We aimed to assess the impact of several rounds of chemical snail control on the presence and number of *Bulinus* in water bodies of hotspot IUs of the SchistoBreak study on Pemba Island, Tanzania, a setting targeted for elimination of urogenital schistosomiasis.

We found that many water bodies were located in the hotspot IUs of our study area. However, the distribution of water bodies containing *Bulinus* was highly heterogeneous, and the only clear predictor for the occurrence of *Bulinus* was a low pH value of the water. Moreover, *Bulinus* were found more often in water bodies with muddy riverbanks, which aligns with results from a study conducted in Ethiopia [19]. Additional, but no unique explanatory factors for *Bulinus* occurrence were reported in other studies from Zanzibar and elsewhere [10, 20–23], making it hard to clearly predict intermediate host snail presence or absence. Overall, only a tiny percentage (0.2%) of snails shed *Schistosoma* cercariae in our study. The very low rate of patent *Schistosoma* infections in intermediate host snails in Pemba is in line with previous studies from the Zanzibar Islands [22, 24, 25]. All these findings point to the very focal transmission of *Schistosoma* and underline the importance of conducting malacological surveys and mapping of snails to decide whether and where niclosamide should be applied [19, 26]. Since niclosamide not only kills snails but also has detrimental effects on other invertebrates, amphibians, and fish, it should be used very carefully and only at foci where intermediate host snails thrive and people are at risk of *Schistosoma* infection [5, 27]. In our study, we found that the use of niclosamide not only has an impact on *Bulinus* but also reduced the number of snails of the genus *Lanistes*. Moreover, while the niclosamide in our study was donated, it needs to be flagged that buying the molluscicide is expensive [28]. Hence, to keep environmental and financial costs at the lowest possible level, snail control with niclosamide should only be conducted at confirmed intermediate host snail habitats, in areas where schistosomiasis is endemic.

Our results clearly show that repeated rounds of snail control effectively reduced the number of *Bulinus* over time, and numerous water bodies became snail-free after the initial application of niclosamide. A decline in the number of intermediate host snails is expected to reduce the risk of *Schistosoma* transmission to humans [6] and hence to support the WHO elimination goals. Indeed, a review of 63 studies on focal snail control indicated that, the longer the duration of snail control, the lower the odds of human *Schistosoma* infection [29]. These findings underscore the impact of snail control on human infection and highlight the need for a prolonged intervention duration for successful schistosomiasis elimination.

The quick recurrence of *Bulinus* in some of the water bodies that were treated with several rounds of niclosamide indicates that the mollusciciding has no enduring impact on *Bulinus* presence and that the snail populations in our study were not fully eliminated. While this suggests that the impact on the ecosystem with a focus on *Bulinus* is not dramatic, it also illustrates that rapidly rebounding snail populations pose a risk of recrudescence of *Schistosoma* transmission and jeopardize the elimination goals.

As recommended by the WHO, for a more sustainable impact on human infections, snail control should be implemented together with other interventions, including treatment with praziquantel, water sanitation and hygiene measures, and behavior change interventions [5]. A combination of interventions not only can result in prevalence reduction but also supports the cost-efficiency of interventions, as shown by simulations from Kenya where adding snail control to school-based MDA was found to be more cost-effective than MDA alone in 95% of the simulations [28]. Ideally, praziquantel treatment interventions are timed to seasonal *Schistosoma* transmission and snail control. Studies showed that snail control is conducted best during the peak transmission season to maximize the impact of molluscicides, while minimizing the frequency of intervention rounds and thereby reducing negative adverse effects on the environment [30–32]. These studies also indicated that MDA is conducted best during the low-transmission season when snail density is at its minimum. However, very likely, neither snail control nor treatment interventions, nor a combination thereof, will result in the complete interruption of *Schistosoma* transmission. To achieve and maintain elimination, people living in endemic areas need to have access to improved water and hygiene facilities to avoid contact with potentially *Schistosoma* cercariae-contaminated freshwater from lakes or streams. Without the requisite infrastructure, a change in human behavior cannot occur. Furthermore, this change in behavior will require that people living in endemic areas understand how *Schistosoma* infections are transmitted, what health consequences an infection can have, and how the infection can be prevented.

Of note, our study has several limitations. First, *Bulinus* are hermaphrodites and a single surviving snail can multiply into a new population. Hence, results on the reduction of snail number categories are to be interpreted with care. Second, the water flow, inflow, and outflow of water bodies may have influenced the effectiveness of mollusciciding owing to rapid dilution of the niclosamide concentration after spraying. Third, snails and cercariae were only identified morphologically at genus level and no molecular analyses were conducted to determine the species. Hence, the very low numbers of *Schistosoma* cercariae found may have been due to infection of *Bulinus* with either *S. haematobium* or *S. bovis*. Determination of the *Schistosoma* species in future studies will be important to shed light on the potential for hybrid species to emerge as a future concern for elimination.

## Conclusions

The distribution of water bodies containing *Bulinus* was very heterogeneous, and the main predictor for *Bulinus* occurrence was low pH of the water. Chemical snail control reduced the number of *Bulinus* in water bodies, particularly when niclosamide was applied in multiple rounds. However, in many water bodies, snails reoccurred after one or multiple treatments. Our results show that chemical snail control can help to reduce snail numbers. However, to fully break the *S. haematobium* life cycle, timely diagnosis and treatment of infected humans, improved access to clean water and sanitation, and increased health literacy of people living in endemic areas remain of prime importance.

## Supplementary Information

Supplementary Material 1: Table 1: Environmental factors associated with *Bulinus* presence in water bodies in the north of Pemba, Tanzania (PDF).

## Data Availability

Data are provided within the manuscript or supplementary information files.

## Abbreviations

CI: Confidence interval
IU: Implementation unit
MDA: Mass drug administration
OR: Odds ratio
PHL-IdC: Public Health Laboratory–Ivo de Carneri
WHO: World Health Organization
