# Supplementary material for: Impact of chemical snail control on intermediate host snail populations for urogenital schistosomiasis elimination in Pemba, Tanzania: findings of a 3-year intervention study
Source: Parasit Vectors. 2024 Nov 26;17:489. doi: 10.1186/s13071-024-06565-2 (PMC11590614; doi:10.1186/s13071-024-06565-2)
Supplement: Supplementary file 1 — Supplementary Material 1: Table 1: Environmental factors associated with Bulinus presence in water bodies in the north of Pemba, Tanzania (PDF). [file 13071_2024_6565_MOESM1_ESM.pdf]

Supplementary File 1

Table 1: Environmental factors associated with *Bulinus* presence in water bodies in the north of Pemba, Tanzania

Univariable and Multivariable Logistic Regression Results

**Covariate**	**Odds Ratio**	**Lower CI**	**Upper CI**	**P-Value**	**Model**	**N**	***Bulinus* present (%)**
WB Size: 5-10 meters	1.58	0.63	4.02	3.29e-01	Univariable	26	50.00
WB Size: >10 meters	1.11	0.36	3.29	8.54e-01	Univariable	17	41.18
WB Depth: 10-50 centimeters	2.00	0.42	14.53	4.20e-01	Univariable	39	33.33
WB Depth: 50-100 centimeters	4.57	0.95	33.79	8.11e-02	Univariable	30	53.33
WB Depth: >1 meter	4.00	0.81	30.02	1.16e-01	Univariable	26	50.00
WB Sediment: Muddy and sandy	1.14	0.28	5.70	8.63e-01	Univariable	80	36.25
WB Sediment: Muddy	6.00	1.08	41.51	4.97e-02*	Univariable	16	75.00
WB Shore: Gradual	1.09	0.41	2.83	8.67e-01	Univariable	30	36.67
WB Shore: Steep	2.34	0.78	7.31	1.33e-01	Univariable	18	55.56
WB Shore: Very steep	3.28	0.86	14.16	8.92e-02	Univariable	11	63.64
Rice field: Separately	3.20	0.86	13.29	9.24e-02	Univariable	21	57.14
Rice field: Partly	1.72	0.56	6.04	3.62e-01	Univariable	55	41.82
Rice field: Fully	1.20	0.23	5.98	8.22e-01	Univariable	12	33.33
Floating vegetation	1.00	0.42	2.34	9.97e-01	Univariable		
Overhanging vegetation	0.61	0.24	1.51	2.97e-01	Univariable		
Non-permanent WB	0.55	0.25	1.21	1.43e-01	Univariable	47	34.04
Log Temperature	0.12	0.00	15.13	4.21e-01	Univariable		
pH	0.37	0.18	0.69	2.92e-03**	Univariable		

**Covariate**	**Odds Ratio**	**Lower CI**	**Upper CI**	**P-Value**	**Model**	**N**	***Bulinus* present (%)**
Conductivity	1.00	1.00	1.00	2.54e-01	Univariable		
Lanistes present	2.08	0.90	5.02	9.13e-02	Univariable		
Lymnaea present	0.92	0.12	5.79	9.30e-01	Univariable		
Thiara present	2.16	0.34	16.94	4.11e-01	Univariable		
WB Sediment: Muddy and sandy	3.23	0.46	30.65	2.66e-01	Multivariable	80	36.25
WB Sediment: Muddy	46.63	4.49	729.51	2.73e-03**	Multivariable	16	75.00
Rice field: Separately	1.22	0.25	6.15	8.06e-01	Multivariable	21	57.14
Rice field: Partly	0.47	0.10	2.24	3.37e-01	Multivariable	55	41.82
Rice field: Fully	0.08	0.01	0.78	4.05e-02*	Multivariable	12	33.33
Floating vegetation	0.42	0.12	1.37	1.63e-01	Multivariable		
Overhanging vegetation	0.29	0.07	1.01	6.50e-02	Multivariable		
pH	0.18	0.07	0.44	3.66e-04***	Multivariable		
Conductivity	1.00	1.00	1.01	7.60e-02	Multivariable		
Lanistes present	6.53	2.02	25.35	3.26e-03**	Multivariable		

Note: * $p < 0.05$, ** $p < 0.01$, *** $p < 0.001$
